# Fulminant Cardiac Sarcoidosis Successfully Treated With Aggressive Immunosuppressive Therapy

**DOI:** 10.1155/cric/1350557

**Published:** 2025-03-11

**Authors:** Kaori Yasumura, Fusako Sera, Yasuhiro Akazawa, Kei Nakamoto, Makiko Kawai, Masako Kurashige, Daisuke Nakamura, Takafumi Oka, Isamu Mizote, Eiichi Morii, Tomohito Ohtani, Yasushi Sakata

**Affiliations:** ^1^Department of Cardiovascular Medicine, Osaka University Graduate School of Medicine, Suita, Japan; ^2^Department of Pathology, Osaka University Graduate School of Medicine, Suita, Japan

**Keywords:** cardiac sarcoidosis, case report, fulminant, giant cell myocarditis, immunohistochemical staining, immunosuppressive therapy, mechanical circulatory support

## Abstract

**Background:** The clinical course of cardiac sarcoidosis is typically subacute, and fulminant cases requiring mechanical circulatory support are rare. Here, we report the case of a patient with pathologically diagnosed cardiac sarcoidosis who presented with fulminant myocarditis and whose cardiac function was improved by aggressive immunosuppressive therapy based on the treatment of giant cell myocarditis.

**Case Presentation:** A 55-year-old woman presented with progressive dyspnoea and nausea that persisted for 1 month and was eventually diagnosed with acute heart failure. Echocardiography showed a reduced left ventricular ejection fraction with thinning of the basal septal wall. During hospitalisation, she experienced ventricular tachycardia and fibrillation attacks, and bradycardia due to a complete atrioventricular block and sinus dysfunction was observed after starting amiodarone. Subsequently, she underwent intra-aortic balloon pump insertion in addition to inotropic agent administration; however, venoarterial extracorporeal membrane oxygenation and Impella 5.0 were needed because biventricular dysfunction progressed. We diagnosed our patient with cardiac sarcoidosis based on the pathological findings revealing inflammatory cell infiltration, including giant cells with extensive fibrosis and granulomas. However, the possibility of giant cell myocarditis could not be ruled out because of the fulminant clinical course; therefore, aggressive immunosuppressive therapy with corticosteroids and cyclosporine was started. Her cardiac function improved, and all mechanical circulatory support and inotropic agents were discontinued.

**Conclusion:** Cardiac sarcoidosis is difficult to differentiate from giant cell myocarditis because they have many similarities in terms of myocardial histopathology and clinical manifestations. While whether the two diagnoses are parts of a single-disease continuum remains debatable, aggressive combination immunosuppressive therapy may contribute to favourable outcomes.

## 1. Introduction

Sarcoidosis is a multisystem granulomatous disease involving various organs. Cardiac lesions (cardiac sarcoidosis (CS)) have three principal manifestations: conduction abnormalities, ventricular arrhythmias, and heart failure [1]. Its clinical course is typically subacute, with rare cases of fulminant myocarditis requiring mechanical circulatory support (MCS). Giant cell myocarditis (GCM), a well-known cause of fulminant myocarditis [2], has been attributed to T-lymphocyte–mediated autoimmune myocardial injury along with CS [3]. These two conditions have many similarities in myocardial histopathology and clinical presentation, leading to the discussion that these two conditions may be part of a single-disease continuum [3–5]. Herein, we report a rare case of a patient with pathologically diagnosed CS presenting with fulminant myocarditis, whose cardiac function was improved by aggressive immunosuppressive therapy based on the treatment of GCM.

## 2. Case Presentation

A 55-year-old woman presented to a previous hospital with progressive dyspnoea and nausea that had persisted for 1 month. Echocardiography showed a reduced left ventricular (LV) ejection fraction (LVEF) of 25%, and she was admitted with a diagnosis of heart failure. During hospitalisation, she developed a complete atrioventricular block when she started taking beta-blockers, vasodilators, and diuretics. After the discontinuation of beta-blockers, atrioventricular conduction was restored; however, administration of an intravenous inotropic agent was required because of persistent symptoms due to low cardiac output; thus, she was transferred to our hospital 14 days after admission to the previous hospital.

At the time of transfer, she presented with mild dyspnoea on exertion and mild limb oedema, with a heart rate of 81 bpm and blood pressure of 98/68 mmHg on inotropic support. Blood test results showed normal hepatic and renal function, except for a mild increase in *γ*-glutamyl transpeptidase levels. Her brain natriuretic peptide (BNP) level was elevated at 287.5 pg/mL, whereas the Troponin T level was within the normal range at 0.015 ng/mL. Lysozyme, Interleukin-2 receptor, and angiotensin-converting enzyme levels were also within normal limits (Table 1).

**Figure 1:**
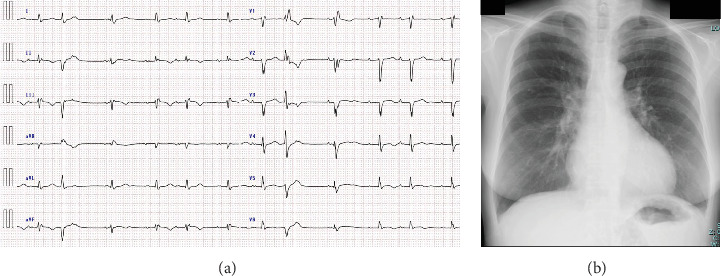
Electrocardiogram and chest radiography recorded at the time of transfer to our hospital. (a) The electrocardiogram reveals first-degree atrioventricular block and frequent premature atrial and ventricular contractions. (b) Chest radiography reveals mild pulmonary oedema and cardiomegaly.

**Figure 2:**
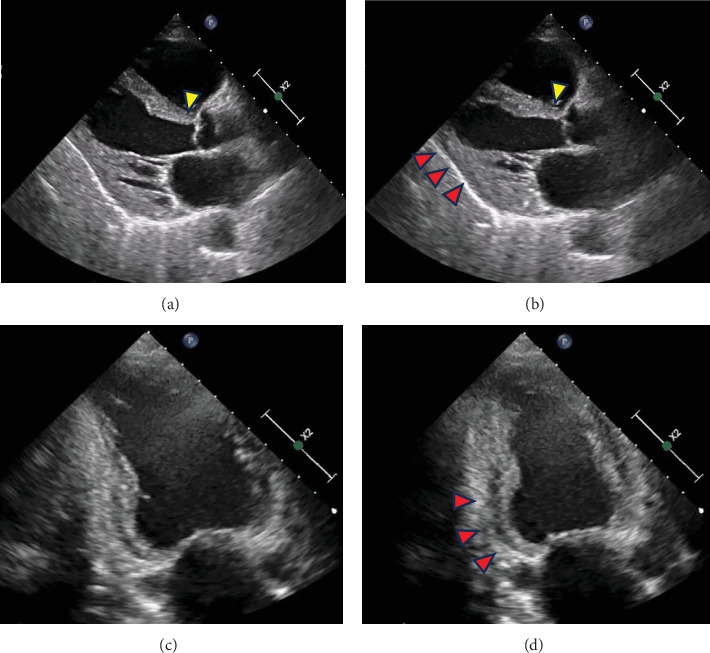
Echocardiography conducted at the time of transfer to our hospital. The echocardiography reveals thinning of the basal septal wall thickness (yellow arrowheads) and severe hypokinesis in the mid-posterior wall (red arrowhead) at (a) end-diastole and (b) end-systole and severe hypokinesis in the basal inferior wall (red arrowhead) at (c) end-diastole and (d) end-systole.

**Figure 3:**
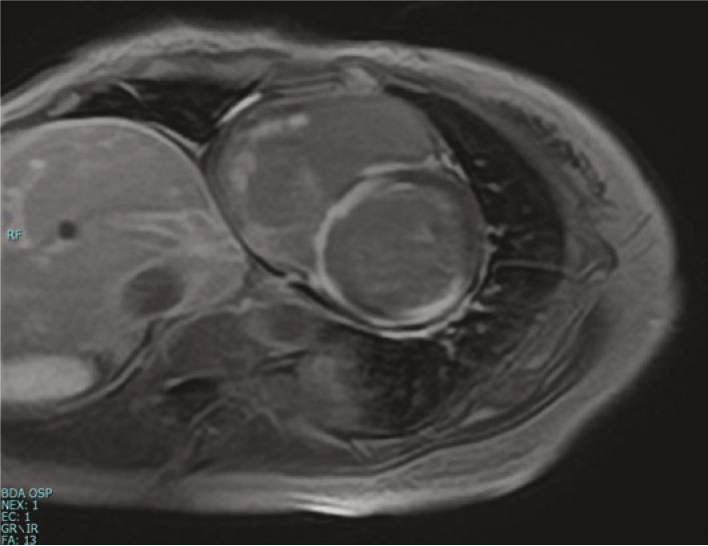
Cardiac magnetic resonance imaging. The image reveals significant late gadolinium enhancement in the basal septal to the inferior wall of the LV and the RV septal wall.

Electrocardiography revealed sinus rhythm with first-degree atrioventricular block, poor R-wave progression, and frequent premature atrial and ventricular contractions (Figure 1(a)). Chest radiograph revealed mild pulmonary oedema and cardiomegaly (Figure 1(b)), and echocardiography revealed an LV end-diastolic diameter of 50 mm and an LVEF of 33% according to the modified Simpson's method. LV wall motion showed generalised hypokinesis, particularly in the basal inferior and mid-posterior walls, with thinning of the basal septal wall (Figures 2(a), 2(b), 2(c), and 2(d), Supporting Information 1, 2, and 3: Videos S1–S3). No significant valvular regurgitations were observed, and right ventricular (RV) contractions were mildly reduced. Cardiac magnetic resonance imaging revealed late gadolinium enhancement (LGE) from the basal septal to the inferior wall of the LV and RV septal wall (Figure 3). Based on the clinical course and findings, CS was suspected, and an endomyocardial biopsy was planned.

Precisely 6 days after the transfer, the patient developed ventricular tachycardia (VT) and fibrillation, and intravenous amiodarone was initiated. The following day, she developed bradycardia with complete atrioventricular block and sinus dysfunction, requiring the insertion of a temporary pacemaker. Coronary angiography revealed no significant stenosis. Right heart catheterisation revealed a very low cardiac index of 1.1 L/min/m^2^, mixed venous oxygen saturation (SvO_2_) of 52%, high pulmonary artery wedge pressure (PAWP) of 18 mmHg, and right atrial pressure (RAP) of 13 mmHg at 80 bpm ventricular pacing, and MCS with intra-aortic balloon pumping (IABP) was initiated.

**Figure 4:**
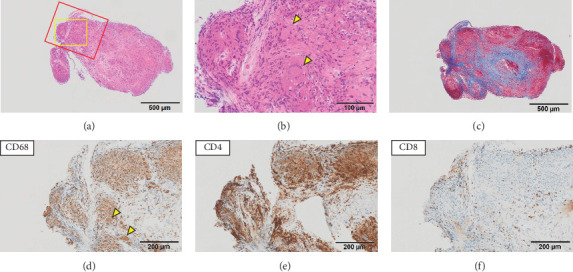
Endomyocardial biopsy results. The specimen shows noncaseating epithelioid granulomas and fibrosis. (a, b) Haematoxylin and eosin staining. (b) is an enlarged image of the inside of a yellow square in (a); yellow arrowheads indicate giant cells. (c) Masson trichrome staining. Images of immunohistochemical staining for the markers of (d) CD68, (e) CD4, and (f) CD8, corresponding to the area indicated by the red square in (a). Giant cells (yellow arrowheads) and epithelioid cells that form granulomas are positive for CD68. CD4 lymphocytes are predominantly accumulated in granulomas, and CD8 lymphocytes are found sporadically at the periphery of granulomas.

Seven endomyocardial biopsy specimens were collected from the right interventricular septum. Pathological findings in the specimens were heterogeneous; five specimens were almost normal, whereas two specimens demonstrated inflammatory cell infiltration, including giant cells with granulomas and fibrosis, suggestive of CS (Figures 4(a), 4(b), and 4(c)). Furthermore, immunohistochemical staining also demonstrated features of CS; granuloma-forming giant cells and epithelioid cells were positive for CD68, and more CD4 lymphocytes were recruited than CD8 lymphocytes. CD4 lymphocytes predominantly accumulated in the granulomas, whereas CD8 lymphocytes were found sporadically only at the periphery of the granulomas (Figures 4(d), 4(e), and 4(f)). However, because the possibility of GCM could not be ruled out due to the fulminant clinical course, immunosuppressive therapy in accordance with GCM treatment was started on the 9th day with 1000 mg/day of methylprednisolone for 3 days and cyclosporine at a target blood level of 160–180 ng/mL. After steroid pulse therapy, methylprednisolone was replaced with oral prednisolone at a dose of 50 mg/day.

**Figure 5:**
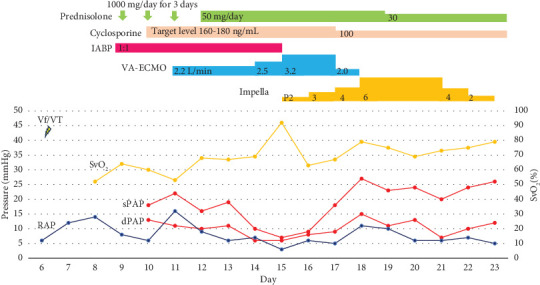
Hemodynamic parameters during mechanical circulatory support and immunosuppressive therapy. Mechanical circulatory support (MCS) with V-A ECMO and Impella 5.0 was initiated because of worsening biventricular function. The combination of immunosuppressive therapy and adequate cardiac unloading with MCS resulted in the improvement of cardiac function, and the patient was successfully weaned from MCS. IABP, intra-aortic balloon pumping; V-A ECMO, venoarterial extracorporeal membrane oxygenation; RAP, right atrial pressure; sPAP, systolic pulmonary artery pressure; dPAP, diastolic pulmonary artery pressure; SvO_2_, mixed venous oxygen saturation; Vf/VT, ventricular fibrillation/ventricular tachycardia.

Under IABP support, SvO_2_ temporarily improved to 64% but subsequently dropped to 53%, and the RAP increased to 16 mmHg. Therefore, we initiated venoarterial extracorporeal membrane oxygenation (V-A ECMO) on Day 11. However, the patient's biventricular dysfunction progressed; the aortic valve opening disappeared, prompting IABP to upgrade to Impella 5.0 on Day 15. Amiodarone was discontinued the same day as ventricular arrhythmias were no longer observed. We prioritised RV unloading with V-A ECMO for circulation management. RV function was restored first, allowing for the successful removal of V-A ECMO on Day 18, with RAP well-controlled at 5 mmHg. Subsequently, LV function gradually improved. By Day 21, she recovered from ventricular pacing dependence to her own sinus rhythm, with a heart rate of over 60 bpm. She was weaned from Impella on Day 23 because SvO_2_ stabilised at 79% and diastolic pulmonary artery pressure was 12 mmHg, even at performance level P2 of Impella. No complications occurred during MCS (Figure 5).

The oral prednisolone dose was carefully tapered to 15 mg/day, and the target blood cyclosporine level was lowered to 100 ng/mL. At discharge, 83 days after transfer to our hospital, her New York Heart Association classification was Class II, and her BNP level was 225.9 pg/mL. Despite starting carvedilol to prevent ventricular arrhythmia, her electrocardiography showed sinus rhythm with only a first-degree atrioventricular block. Echocardiography demonstrated LVEF recovery to 48%, with asynergy at the thinning of the basal septal, basal inferior, and mid-posterior walls. Owing to residual nonsustained VT, she was discharged home on a wearable cardioverter defibrillator and implanted with a subcutaneous implantable cardioverter-defibrillator after tapering prednisone. One year after discharge, the patient continued immunosuppressive therapy with oral prednisolone at a dose of 10 mg/day and a maintained blood cyclosporine level of approximately 80 ng/mL. Echocardiography revealed an LVDd of 49 mm and an LVEF of 48%, with noticeable thinning of the basal septal wall; however, LV wall motion in other areas had improved. The patient remained in New York Heart Association Class II, demonstrating stable functional status without recurrence of heart failure or arrhythmias. Additionally, gallium scintigraphy showed no myocardial accumulation.

## 3. Discussion

In this report, we describe a case of CS presenting with fulminant myocarditis that developed into severe biventricular failure requiring MCS, which was successfully treated with aggressive immunosuppressive therapy.

Sarcoidosis is a multisystem, granulomatous disease of unknown aetiology with a relatively high frequency of cardiac involvement; cardiac lesions are found in approximately 25% of autopsy cases of sarcoidosis [6, 7]. LGE on magnetic resonance imaging is detected at a similar frequency in patients with sarcoidosis [8, 9]. The clinical manifestations of cardiac lesions vary depending on the location and degree of granulomatous inflammation, and their clinical course is usually slowly progressive and subacute. Mild cardiac lesions are often asymptomatic, and symptoms appear when granulomatous lesions disrupt the conduction system or cardiac pump function [1]. Our patient exhibited all the main manifestations of CS, including atrioventricular block, VT, and decompensated heart failure. However, her clinical course was rapid and fulminant, with severe biventricular dysfunction, suggesting that granulomatous inflammation extensively involved both ventricles. Despite the severity of her clinical course, the troponin level at the time of transfer was unexpectedly within the normal range, suggesting that troponin may have limited sensitivity for the early diagnosis of fulminant CS.

GCM is a well-known cause of fulminant myocarditis, and its pathological findings are similar to those of CS in some respects. For instance, giant cells are present in equivalent numbers in both GCM and CS [5]. Notably, there is no consensus among pathologists regarding their respective diagnostic criteria [10]. The presence of noncaseating epithelioid granulomas is the gold standard for CS diagnosis; however, the interpretation of the presence of granulomas in GCM is controversial. Davies, Pomerance, and Teare emphasised the absence of granulomas in GCM [11], which was adopted in a landmark report by a multicentre GCM Study Group in 1997 [12]. However, Okura et al. [5] stated that, while granulomas are rarely present in GCM, their presence does not rule out GCM if the degree of necrosis is not proportional to the degree of granulomatous inflammation. They also showed distinctions in the pathology of CS and GCM; in their study, granulomas and fibrosis were more frequent in the CS group, whereas eosinophils, myocyte damage, and foci of lymphocytic myocarditis were more frequent in the GCM group [5].

CS and GCM have been attributed to T-lymphocyte–mediated autoimmune myocardial injury, and immunohistochemical staining is useful in differentiating these two conditions [13]. The lymphocytes in the inflammatory infiltrates of GCM are predominantly CD8 positive, whereas those in CS are predominantly CD4 positive [13]. Furthermore, the localisation of CD4 lymphocytes, predominantly in granulomas, and CD8 lymphocytes, sporadically at the periphery of granulomas, is a pathological feature of CS [14]. We diagnosed our patient with CS based on the pathological findings of extensive fibrosis and granulomas, as well as the immunohistochemistry findings of CD4-positive lymphocyte predominance and localisation of CD4 and CD8 lymphocytes.

However, the clinical course of our patient was rapid and fulminant, requiring MCS, indicating similarities of the condition with GCM. While cases of fulminant CS, such as ours, have been reported previously, the reported clinical outcomes varied depending on the severity of cardiac dysfunction and treatment [15–19]. Two patients who required MCS due to severe LV dysfunction but had preserved RV function were treated with corticosteroids alone or with corticosteroids and cyclosporine and had good outcomes [15, 16]. Chakrala et al. reported a CS case with severe biventricular heart failure and cardiogenic shock successfully treated with IABP and aggressive immunosuppressive therapy [17]. Two other patients presented with severe biventricular dysfunction requiring biventricular MCS, in which the cardiac function did not recover, and orthotopic heart transplantation was performed [18, 19]. In both cases, CS was diagnosed by pathological examination of the explanted hearts, and steroid pulse therapy was attempted in only one case prior to heart transplantation [18]. To our knowledge, this is the first report of a fulminant CS case requiring biventricular MCS with a good clinical outcome after aggressive immunosuppressive therapy.

Corticosteroids are the first-line immunosuppressants for patients with CS [14, 20], whereas a combination therapy with steroids and other immunosuppressants, such as cyclosporine, is recommended for patients with GCM [12, 21, 22]. Cyclosporine effectively suppresses T-cell–mediated diseases by inhibiting T-cell activation through the blockade of Interleukin-2 production in T-cells [23]. The efficacy of cyclosporine in treating GCM has been demonstrated in both retrospective and prospective clinical studies [12, 24], as well as in an animal model of autoimmune GCM [25]. CS and GCM share a common background of T-lymphocyte–mediated autoimmune myocardial injury. In our case of fulminant CS, we implemented potent immunosuppressive therapy combining corticosteroids with cyclosporine, which led to significant recovery of cardiac function. This clinical response suggests that the fulminant progression of CS may share overlapping mechanisms with the pathophysiology of GCM. However, whether CS and GCM represent distinct entities or phenotypes of a single T-cell–mediated inflammatory cardiomyopathy remains an area of active investigation. Ekström et al. showed that in 62% of cases previously diagnosed with GCM, granulomas were identified inside or outside the heart and were considered CS lesions [3]. Nordenswan et al. reported that GCM presented more advanced heart failure and a worse long-term prognosis compared to CS. However, they found that prognosis was determined by the extent of myocardial injury rather than the histopathological diagnosis of GCM or CS, suggesting that these conditions may represent severity phenotypes of a single disease [26]. Conversely, Lassner et al. conducted gene expression profiling and identified significant differences in the myocardial expression of some genes between GCM and CS, suggesting that they may be distinct clinical entities [27]. Further research is warranted to clarify these findings. Given the difficulty in definitively excluding GCM and the potential involvement of similar pathological mechanisms, especially in fulminant CS, we recommend aggressive immunosuppressive therapy, similar to that used for GCM, as a treatment strategy for fulminant CS.

In conclusion, our case highlights the similarities between CS and GCM and the possibility that some CS cases benefit from aggressive immunosuppressive therapy. Further studies are needed to elucidate the mechanisms underlying the development of fulminant heart failure in CS cases and also to identify high-risk cases.

## Figures and Tables

**Table 1:** Laboratory results at the time of transfer to our hospital.

**Laboratory variables**	**Value**	**Reference range**
White blood cell count (10^3^/*μ*L)	5.0	3.3–9.4
Haemoglobin (g/dL)	16.2	12.0–15.0
Platelet count (10^4^/*μ*L)	19.5	13.0–32.0
AST (IU/L)	25	< 40
ALT (IU/L)	31	< 40
GGT (IU/L)	95	8–51
Urea (mg/dL)	13	7–22
Creatinine (mg/dL)	0.52	0.5–0.9
Sodium (mEq/L)	141	138–145
Potassium (mEq/L)	4.0	3.6–4.8
Albumin (g/dL)	4.0	3.6–4.7
C-reactive protein (mg/dL)	0.12	0.0–0.2
BNP (pg/mL)	287.5	< 40
Troponin T (ng/mL)	0.015	< 0.100
Lysozyme (*μ*g/mL)	7.3	5.0–10.0
Interleukin-2 receptor (U/mL)	317	121–613
ACE (IU/L)	11.0	7.7–29.4

Abbreviations: ACE, angiotensin-converting enzyme; ALT, alanine aminotransferase; AST, aspartate aminotransferase; BNP, brain natriuretic peptide; GGT, *γ*-glutamyl transpeptidase.

## Data Availability

All data, including laboratory values, imaging files, and other raw data, were obtained from the patient's medical records and can be made available in a deidentified format.

## Supporting Information

Supporting Information 1 Video S1: Parasternal long-axis echocardiographic view at the time of transfer.

Supporting Information 2 Video S2: Apical four-chamber echocardiographic view at the time of transfer.

Supporting Information 3 Video S3: Apical two-chamber echocardiographic view at the time of transfer.
